# An antibody against Siglec-15 promotes bone formation and fracture healing by increasing TRAP^+^ mononuclear cells and PDGF-BB secretion

**DOI:** 10.1038/s41413-021-00161-1

**Published:** 2021-11-01

**Authors:** Gehua Zhen, Yang Dan, Ruomei Wang, Ce Dou, Qiaoyue Guo, Melissa Zarr, Linda N. Liu, Lieping Chen, Ruoxian Deng, Yusheng Li, Zengwu Shao, Xu Cao

**Affiliations:** 1 Department of Orthopedic Surgery, Institute of Cell Engineering, Johns Hopkins University School of Medicine, Baltimore, MD USA; 2 Department of Orthopedics, Union Hospital, Tongji Medical College, Huazhong University of Science and Technology, Wuhan, China; 3 NextCure, Inc., Beltsville, MD, USA; 4 Department of Immunobiology, Yale University School of Medicine, New Haven, CT USA; 5 Department of Biomedical Engineering, Johns Hopkins University School of Medicine, Baltimore, MD USA

**Keywords:** Diseases, Osteopetrosis

## Abstract

Osteoporosis (OP) is a common age-related disease characterized by a deterioration of bone mass and structure that predisposes patients to fragility fractures. Pharmaceutical therapies that promote anabolic bone formation in OP patients and OP-induced fracture are needed. We investigated whether a neutralizing antibody against Siglec-15 can simultaneously inhibit bone resorption and stimulate bone formation. We found that the multinucleation of osteoclasts was inhibited in *SIGLEC-15* conditional knockout mice and mice undergoing Siglec-15 neutralizing antibody treatment. The secretion of platelet-derived growth factor-BB (PDGF-BB), the number of tartrate-resistant acid phosphatase-positive (TRAP^+^) mononuclear cells, and bone formation were significantly increased in the *SIGLEC-15* conditional knockout mice and antibody-treated mice. The anabolic effect of the Siglec-15 neutralizing antibody on bone formation was blunted in mice with *Pdgfb* deleted in TRAP^+^ cells. These findings showed that the anabolic effect of the Siglec-15 neutralizing antibody was mediated by elevating PDGF-BB production of TRAP^+^ mononuclear cells. To test the therapeutic potential of the Siglec-15 neutralizing antibody, we injected the antibody in an ovariectomy-induced osteoporotic mouse model, which mimics postmenopausal osteoporosis in women, and in two fracture healing models because fracture is the most serious health consequence of osteoporosis. The Siglec-15 neutralizing antibody effectively reduced bone resorption and stimulated bone formation in estrogen deficiency-induced osteoporosis. Of note, the Siglec-15 neutralizing antibody promoted intramembranous and endochondral ossification at the damaged area of cortical bone in fracture healing mouse models. Thus, the Siglec-15 neutralizing antibody shows significant translational potential as a novel therapy for OP and bone fracture.

## Introduction

Siglec-15 is a member of the sialic acid-binding immunoglobulin-type lectin (Siglecs) family, a group of cell-surface receptors that potentially regulate the adaptive immune response via cell–cell interactions with target cells through their sialic acid-binding N-terminal Ig-like V-type domain. Unlike most other Siglec family members, Siglec-15 has been conserved throughout vertebrate evolution and is expressed primarily by macrophages and dendritic cells.^1^ Previous studies have shown that the expression of Siglec-15 is upregulated in mature osteoclasts.^2,3^ Knocking down the expression of Siglec-15 impaired the receptor activator of nuclear factor kappa-B ligand (RANKL)-induced differentiation of osteoclasts. Interestingly, Siglec-15 is highly expressed in giant cell tumors of bone, which are characterized by abundant osteoclast-like cells with lucent lesions in the epiphyseal region of bone and subsequent fracture.^2^ These data suggest that Siglec-15 is a critical factor in osteoclast formation. Indeed, previous research found that *SIGLEC-15-*null mice are resistant to estrogen deficiency-induced bone loss.^4^ The reduction in osteoclastogenesis that potentially contributes to the significantly reduced bone resorption observed in *SIGLEC-15*-deficient mice is likely mediated by the Siglec-15-DAP12-Syk signaling cascade.^5,6^ Notably, the osteoclasts in the *SIGLEC-15-*null mice were small and failed to spread on the bone surface,^4^ indicating that Siglec-15 is involved in the multinucleation and maturation of osteoclasts.

Osteoporosis is a common age-related disease characterized by deterioration of bone mass and structure that predisposes patients to fragility fractures. Osteoporosis affects more than 20 million people in the U.S. annually. Worldwide, osteoporosis induces 8.9 million fractures, constituting a major social and economic health burden.^7^ Current mainstay U.S. Food and Drug Administration (FDA)-approved treatments for osteoporosis can be assigned to one of two categories: (1) antibone-resorptive treatments, which decrease bone resorption and/or inhibit bone turnover, and (2) anabolic treatments, which stimulate new bone formation. Bisphosphonates are the “first-line” antiresorptive osteoporosis medications and are frequently prescribed in clinics. With the widespread introduction of bisphosphonates into clinical practice, adverse effects, such as osteonecrosis of the jaw, increased risk of esophageal cancer, and severe suppression of bone turnover, have been recognized.^8–10^ Long-term use of bisphosphonates impaired the bone-forming capacity and even blunted the anabolic effect of parathyroid hormone (PTH) treatment.^11^ PTH is the only FDA-approved anabolic osteoporosis medication. Intermittent administration of PTH increases bone mass and strength.^12^ However, because of the potential risk of inducing osteosarcoma and the high cost, PTH use has been limited to 2 years and is considered a “second-line” medication reserved for patients with severe osteoporosis at high risk of fracture.^13^ The use of other antiresorptive osteoporosis medications is limited by their adverse effects. Denosumab (Prolia^®^) is a human monoclonal antibody that has beneficial effects in treating patients with severe osteoporosis, particularly those with a high risk of fracture.^14^ However, patients taking denosumab frequently reported trouble breathing, backache, and pain in muscles or bones.^15^ Possibly because the sole use of either antiresorptive or anabolic OP drugs leads to imperfect clinical results, many studies have evaluated the effect of PTH together with antiresorptive agents.^16,17^ However, concomitant use of alendronate (a classic bisphosphonate) and teriparatide (a recombinant fragment of PTH) did not elevate bone mass above that of PTH treatment only.^18^ Moreover, denosumab treatment followed by teriparatide treatment paradoxically triggered a progressive reduction in bone loss, particularly in the lumbar spine and proximal femur.^19^

The primary causes of bone loss in OP patients are increased osteoclast activity and/or decreased bone formation. Antiresorptive and anabolic drugs each target one mechanism. A dual-function medication targeting both mechanisms may achieve a superior effect in combating bone loss, but no such medication is currently available. Mature osteoclasts, multinuclear cells generated from the fusion of tartrate-resistant acid phosphatase-positive (TRAP^+^) mononuclear cells,^20^ are the primary cells for bone resorption. A previous study showed that TRAP^+^ mononuclear cells can promote bone formation by secreting platelet-derived growth factor-BB (PDGF-BB).^21^ TRAP^+^ mononuclear cells are the major source of PDGF-BB, which induces type H vessel (CD31^hi^Emcn^hi^) formation, in the bone marrow.^21^ Recent studies have shown that type H vessels are essential for new bone formation because osteogenesis is always coupled with angiogenesis.^22,23^ Bone resorption efficiency was shown to be linearly correlated with the number of nuclei in osteoclasts.^24^ Therefore, pharmacological treatments that can halt the maturation process of osteoclasts and increase the number of TRAP^+^ mononuclear cells hold promise as novel therapies for treating osteoporosis.

Siglec-15 is expressed primarily in macrophage and monocyte lineage cells and is implicated in osteoclastogenesis. In our study, we conditionally knocked out *SIGLEC-15* in myeloid lineage cells and investigated the role of Siglec-15 in bone formation. We investigated whether the maintenance of TRAP^+^ cells at the mononuclear stage underlies the effect of Siglec-15 on bone formation. We found that suppressing Siglec-15 promotes the secretion of PDGF-BB in bone marrow by increasing the number of TRAP^+^ mononuclear cells. To test the therapeutic potential of the Siglec-15 neutralizing antibody, we first applied the antibody in an ovariectomy-induced osteoporotic mouse model, which mimics postmenopausal osteoporosis in women. Fracture is the most serious health consequence of osteoporosis; therefore, we also tested whether the Siglec-15 neutralizing antibody could promote fracture healing in two bone healing models. The results showed that the Siglec-15 neutralizing antibody increases bone formation in estrogen deficiency-induced osteoporosis and fracture healing mouse models.

## Results

### Knockout of Siglec-15 in macrophage-lineage cells increased TRAP^+^ mononuclear cells and decreased osteoclastic bone resorption

**Fig. 1:**
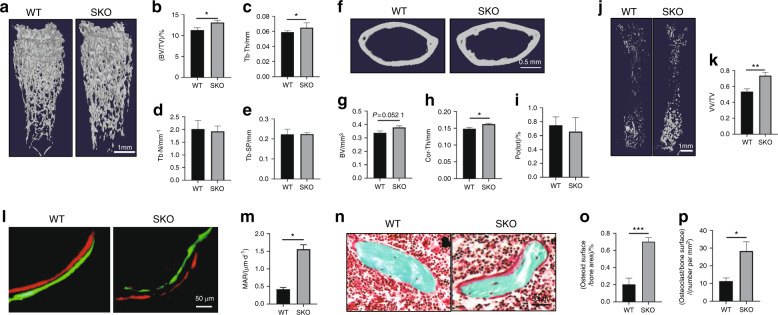
SKO mice exhibited increased bone formation and decreased bone resorption. **a** Representative three-dimensional µCT images of the trabeculae in the femurs of 3-month-old WT and SKO mice. Quantitative analysis of the µCT scan in **a**: **b** BV/TV percentage bone volume, **c** Tb. Th trabecular thickness, **d** Tb. N trabecular number, and **e** Tb. Sp trabecular separation. **f** Representative three-dimensional µCT images of the cortical bone in the femurs of 3-month-old WT and SKO mice. Quantitative analysis of the µCT scan in **f**: **g** BV bone volume, **h** Cor. Th cortical bone thickness, **i** Po (tot) total porosity. **j** Representative three-dimensional reconstructed µCT-based angiography of vessels in mouse femurs. **k** Quantitative analysis of vessel volume in **j**. VV vessel volume, TV tissue volume. **l** Double labeling of the mineral layers in mouse femur trabecular bone. **m** Quantitative analysis of the mineral apposition rate in **l**. **n** Trichrome staining of mouse femurs. The osteoid layer is labeled red. Quantitative analysis of the osteoid-covered surface (**o**) and osteoblast numbers (**p**). **a**–**p** *n* = 5, *P < 0.05, *P < 0.01, and ***P < 0.000 1 Error bar represents the standard error of the mean.

To investigate the role of Siglec-15^+^ osteoclast lineage cells during bone remodeling, we generated LysMCre:Siglec-15^f/f^ (SKO) mice by crossing LysM^Cre^ mice with Siglec-15^flox/flox^ mice. In the SKO mice, the Siglec-15 gene (*SIGLEC-15)* was deleted specifically in lysozyme-expressing cells, the cells that develop into osteoclasts. We used microcomputed tomography (μCT) analysis to examine the overall bone mass and structure of the femurs from 3-month-old SKO mice and compared the results with those of wild-type (WT) mice (Fig. 1a–i). We observed significantly greater bone mass in the femurs of the 3-month-old SKO mice than that of the WT mice, particularly in the secondary spongiosa. Compared with the WT mice, the SKO mice had a greater bone volume to tissue volume ratio (BV/TV) (Fig. 1a, b) and trabecular thickness (Tb. Th) (Fig. 1a, c), whereas trabecular number (Tb. N) (Fig. 1a, d) and trabecular separation (Tb. Sp) (Fig. 1a, e) remained similar between the two groups. The cortical bone volume was slightly higher in the SKO mice than in the WT mice but did not achieve a significant difference (Fig. 1f, g). The thickness of the cortical bone (Cor. Th) significantly increased in the SKO mice relative to the WT mice (Fig. 1f, h). We did not observe a difference in porosity in the cortical bone between these two groups of mice (Fig. 1f, i). CT-based angiography showed that the volume of blood vessels in the bone marrow of the SKO mice was greater than that of the WT mice (Fig. 1j, k). Double labeling showed that the osteoblastic bone formation rate was significantly increased in the SKO mice compared the WT mice (Fig. 1l, m). Trichrome staining showed that the osteoid-covered bone surface was enlarged and that the number of osteoblasts actively producing bone matrix was higher in the SKO mice than in the WT mice (Fig. 1n–p). Our findings show that accelerated bone formation contributes to greater bone mass in the SKO mice than in the WT mice.

**Fig. 2:**
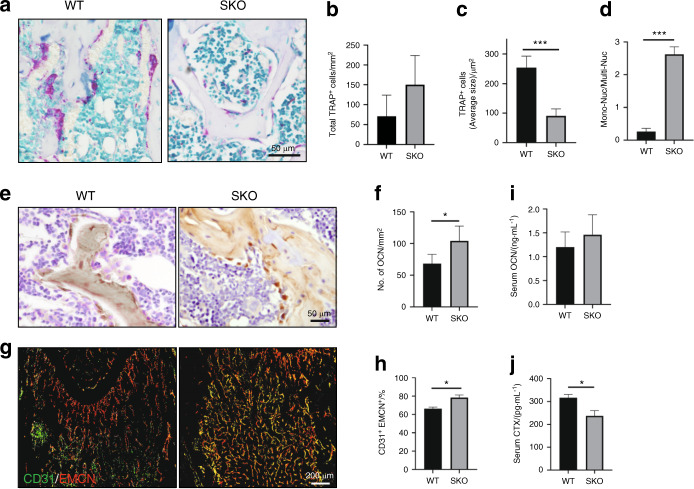
The numbers of mononuclear TRAP^+^ cells, type H vessels, and osteoblasts were increased in the SKO mice. **a** Representative images of TRAP staining of the distal femurs of 3-month-old WT and SKO mice. **b**–**d** Quantitative analysis of the TRAP staining in **a**. The number and size of TRAP^+^ cells were normalized to the bone area. Mon-Nuc: mononuclear TRAP^+^ cells, Multi-Nuc: multinuclear TRAP^+^ cells. *n* = 5, ***P < 0.001. Error bar represents the standard error of the mean. **e** Representative images of immunohistological staining of osteocalcin in the distal femurs of the 3-month-old WT and SKO mice. **f** Quantitative analysis of the osteocalcin staining in **e**. *n* = 5, *P < 0.05. Error bar represents the standard error of the mean. **g** Cofluorescence staining of CD31 (green) and endomucin (EMCN) (red) in mouse femurs. **h** Quantitative analysis of the ratio of CD31 and EMCN double-positive fluorescence intensity in **g**. *n* = 5, *P < 0.05. Error bar represents the standard error of the mean. ELISAs of OCN (**i**) and CTX (**j**) in the serum of the WT and SKO mice. The mice were sacrificed at 3 months of age. *n* = 5, *P < 0.05

To determine whether decreased osteoclast activity also contributes to the bone phenotype we observed in the SKO mice, we performed TRAP staining to characterize the number and location of osteoclasts. We observed no significant difference in the total number of TRAP^+^ cells between the SKO and WT mice (Fig. 2a, b). Compared with the WT mice, the SKO mice had significantly smaller TRAP^+^ cells (Fig. 2a, c) and a significantly higher ratio of TRAP^+^ mononuclear cells (<4 nuclei) to TRAP^+^ multinuclear osteoclasts (≥4 nuclei) (Fig. 2a, d). These findings indicate that Siglec-15 regulates the balance between TRAP^+^ mononuclear cells and multinuclear osteoclasts. Next, we examined whether the increase in TRAP^+^ mononuclear cells led to the augmented bone mass observed in the SKO mice. Immunohistological staining of osteocalcin (OCN) showed that the number of osteoblasts on the bone surface was greater in the SKO mice than in the WT mice (Fig. 2e, f). The number of type H vessels that were double-positive for CD31 and endomucin (EMCN) was significantly greater in the SKO mice than in the WT mice (Fig. 2g, h). Additionally, we conducted ELISAs measuring serum OCN and carboxy-terminal collagen crosslinks (CTX) to examine whether decreased bone resorption or increased bone formation is the primary mechanism that contributes to increased bone mass in the SKO mice. We found that the serum OCN levels were slightly increased in the SKO mice relative to the WT mice but were not significantly different (Fig. 2i). The serum CTX levels were significantly lower in the SKO mice than in the WT mice (Fig. 2j). These data indicate that halted osteoclast maturation impairs osteoclast function in bone resorption and, more importantly, that an increased number of TRAP^+^ mononuclear cells promotes type H vessel and osteoblastic bone formation in the SKO mice.

### PDGF-BB secretion was increased in the bone remodeling environment of the SKO mice

**Fig. 3:**
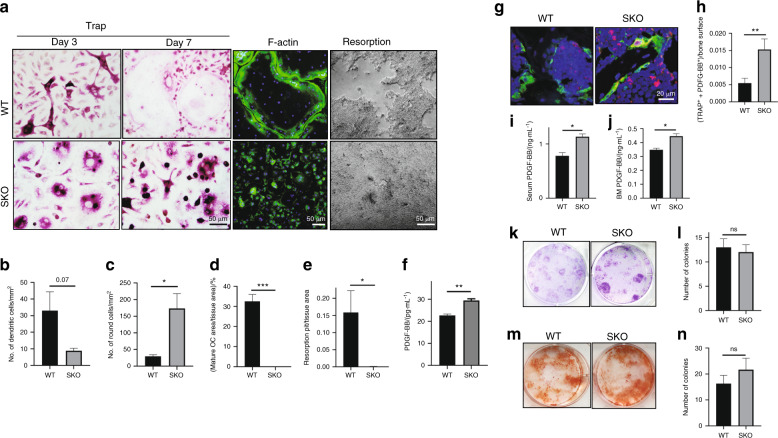
Osteoclast maturation and bone resorption were impaired with excessive secretion of PDGF-BB in the SKO mice. **a** Representative images of TRAP staining (first and second columns), phalloidin staining (third column), and resorption pits (fourth column) of the osteoclast cultures that were induced from primary mouse bone marrow monocytes. TRAP staining was conducted on days 3 and 7 of osteoclastogenic induction. Phalloidin staining and resorption assays were conducted on day 7. **b**, **c** Quantitative analysis of TRAP staining on day 3 of osteoclastogenesis in **a**. **d** Quantitative analysis of TRAP staining on day 7 of osteoclastogenesis in **a**. *P < 0.05, ***P < 0.001. **e** The ratio of the resorption area in **a**. *P < 0.05. **f** ELISAs of the concentration of PDGF-BB in the conditioned media collected on day 7 of osteoclastogenesis. **P < 0.01. **g** Coimmunofluorescence staining of TRAP (green) and PDGF-BB (red) in the trabecular bone of 3-month-old WT or SKO mice. **h** Quantitative analysis of the number of TRAP^+^PDGF-BB^+^ cells in **g**. *n* = 5, **P < 0.01. ELISAs of the concentration of PDGF-BB in the serum (**i**) and bone marrow (**j**) of the 3-month-old WT or SKO mice. *P < 0.05. **k**, **l** Crystal violet staining and quantitative analysis of bone marrow stromal cells cultured for 10 days. **m**, **n** Alizarin red staining of bone marrow stromal cells cultured in osteogenic medium for 21 days. Bone marrow stromal cells were collected from the bone marrow of the 3-month-old WT or SKO mice. ns: not significant

To further characterize the cellular and molecular mechanisms of increased bone formation in the SKO mice, we performed in vitro osteoclastogenesis and bone resorption assays using primary bone marrow monocytes isolated from femurs of the SKO and WT mice. After 3 days of induction with macrophage colony-stimulating factor 1 (mCSF-1) and RANKL, the TRAP^+^ mononuclear cells from the SKO mice had a substantially different morphology than the cells from their WT littermates. Most of the TRAP^+^ cells from the SKO mice were isolated, spherical cells with two or three enlarged nuclei, whereas the WT group had more dendritic-like TRAP^+^ cells with their protrusions connected to each other (Fig. 3a–c). After 7 days of culture with mCSF-1 and RANKL, the TRAP^+^ cells in the WT group fused as multinuclear osteoclasts that covered 30%–40% of the culture surface with clear actin ring formation (Fig. 3a, d). Most TRAP^+^ cells in the SKO group remained mononuclear and rarely fused to form multinuclear osteoclasts (Fig. 3a, d). As expected, the resorption area in the WT group was significantly larger than that of the SKO group (Fig. 3a, e). Importantly, the concentration of PDGF-BB in the conditioned medium of the SKO group was significantly higher than that of the WT group (Fig. 3f). This result was further validated in vivo by coimmunofluorescence staining of PDGF-BB and TRAP in femur sections from the SKO and WT mice. The fluorescence intensity of PDGF-BB in the SKO mice was significantly higher than that in the WT mice. Additionally, PDGF-BB^+^ staining was colocalized with TRAP^+^ cells in the SKO mice, whereas this phenotype was absent in the WT mice (Fig. 3g, h). PDGF-BB levels in both the serum and bone marrow of the SKO mice in enzyme-linked immunosorbent assays (ELISAs) significantly increased relative to those of the WT mice (Fig. 3i, j). Osteoblast differentiation assays in stromal cells collected from the long bone marrow of the WT and SKO mice were not significantly different (Fig. 3k–n), suggesting that increased bone formation in the SKO mice is not caused by the increased number of mesenchymal stromal cells/osteoprogenitors or an elevated intrinsic osteogenic capacity. Therefore, the elevated PDGF-BB secreted by TRAP^+^ mononuclear cells likely contributes to increased anabolic bone formation in the SKO mice.

### Siglec-15 neutralizing antibody treatment increased the number of TRAP^+^ mononuclear cells and bone formation

**Fig. 4:**
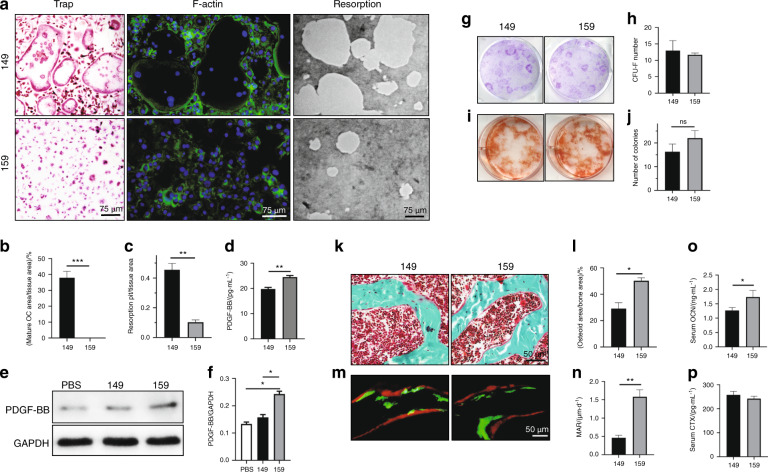
Injection of a Siglec-15 neutralizing antibody halted the maturation of osteoclasts and increased bone formation. **a** Representative images of TRAP staining (first column), phalloidin staining (second column), and resorption pits (third column) of the osteoclast cultures that were induced from primary mouse bone marrow monocytes. All experiments were conducted on day 7 of osteoclastogenic induction. **b**, **c** Quantitative analysis of the TRAP staining and resorption area in **a**. **P < 0.01, ***P < 0.001. **d** ELISAs of the concentration of PDGF-BB in the conditioned media that was collected on day 7 of osteoclastogenesis. **P < 0.01. **e** Western blot of PDGF-BB in the cell lysate of osteoclasts on day 7 of osteoclastogenic induction. **f** Quantitative analysis of blot size in **e**. *P < 0.05. **g**, **h** Crystal violet staining and quantitative analysis of bone marrow stromal cells cultured for 10 days. **i**, **j** Alizarin red staining of bone marrow stromal cells cultured in osteogenic medium for 21 days. Bone marrow stromal cells were collected from 4-month-old mice that received NP-149 or NP-159 injection for 1 month. ns: not significant. **k** Trichrome staining of femurs collected from the mice treated with NP-149 or NP-159 for 2 months. The osteoid layer is labeled red. **l** Quantitative analysis of the osteoid-covered surface. *n* = 5, *P < 0.05. **m** Double labeling of the mineral layers in mouse femur trabecular bone. The mice were treated with NP-149 or NP-159 for 1 months. **n** Quantitative analysis of the mineral apposition rate in **m**. *n* = 5, **P < 0.01. ELISAs of OCN (**o**) and CTX (**p**) in the serum from the mice treated with NP-149 or NP-159 for 1 month. *n* = 5, *P < 0.05

For translational purposes, we then tested whether the Siglec-15 neutralizing antibody could increase the number of TRAP^+^ mononuclear cells for bone formation. First, osteoclastogenesis was induced in primary mouse bone marrow monocytes. After incubation in mCSF-1 for 3 days, the Siglec-15 neutralizing antibody (NP-159) or a control antibody (NP-149) was added to the medium together with RANKL. Similar to the results from the SKO mice, primary monocytes largely remained TRAP^+^ mononuclear cells with NP-159 treatment (Fig. 4a, b). Osteoclast actin ring formation was observed in the NP-149 group but not in the NP-159 group. Again, the resorption assay showed that the resorption area in the NP-149 control group was significantly larger than that of the NP-159 group (Fig. 4a, c). Importantly, PDGF-BB levels were significantly higher in the conditioned medium of the NP-159 group than in that of the NP-149 group, as determined by ELISAs (Fig. 4d). Western blots also showed that the PDGF-BB levels in the lysate of the NP-159-treated cells were significantly higher than those of the NP-149-treated cells (Fig. 4e, f). Osteoblast differentiation assays of the primary stromal cell cultures that were incubated in NP-149 or NP-159 indicated that these cultures were not significantly different (Fig. 4g–j). To validate the findings in vivo, we treated C57B/L6 WT mice with NP-149 or NP-159 twice a week for 1 month. Trichrome staining showed that osteoid surface area and the number of osteoblasts increased significantly in the NP-159-treated mice relative to the NP-149-treated (control) mice (Fig. 4k, l). Double labeling showed that the bone formation rate was significantly higher in the mice subjected to NP-159 treatment (Fig. 4m, n). Interestingly, monocytes collected from the bone marrow of the mice treated with Siglec-15 neutralizing antibodies had an impaired ability to form mature osteoclasts when incubated in osteoclastogenic media for 7 days (Fig. S1). Moreover, ELISAs showed that the serum OCN levels were significantly enhanced in the NP-159-treated mice compared to the NP-149-treated mice (Fig. 4o). In contrast, there was no difference in the serum CTX levels between these two groups of mice (Fig. 4p). These data suggest that the Siglec-15 neutralizing antibody can inhibit osteoclast bone resorption while promoting bone formation.

### Knockout of PDGF-BB in TRAP^+^ lineage cells blunted the effect of the Siglec-15 neutralizing antibody on bone formation

**Fig. 5:**
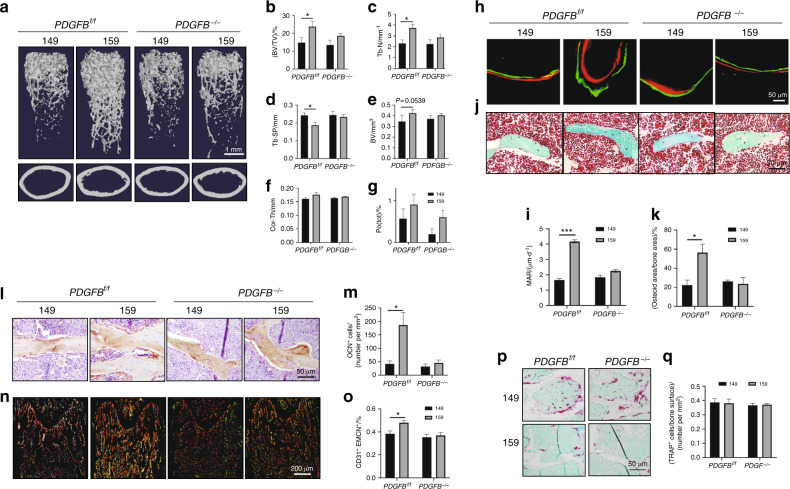
Knockout of PDGF-BB in the osteoclast lineage blunted the effect of the Siglec-15 neutralizing antibody on bone formation. All data in this figure are derived from 5-month-old *PDGFB*^*f/f*^ or *PDGFB*^−/−^ mice treated with NP-149 or NP-159 for 2 months. **a** Representative three-dimensional µCT images of the mouse femurs. Quantitative analysis of the µCT scan in **a**: **b** BV/TV percentage trabecular bone volume, **c** Tb. N trabecular number, **d** Tb. Sp trabecular separation. **e** BV cortical bone volume, **f** Cor. Th cortical bone thickness, and **g** Po (tot) total porosity of cortical bone. *n* = 5, *P < 0.05. **h** Double labeling of the mineral layers in mouse femur trabecular bone. **i** Quantitative analysis of the mineral apposition rate in **h**. *n* = 5, ***P < 0.001. **j** Trichrome staining of mouse femurs. The osteoid layer is labeled red. **k** Quantitative analysis of the osteoid-covered surface in **p**. *n* = 5, *P < 0.05. **l** Representative images of immunohistological staining of osteocalcin in the distal femurs. **m** Quantitative analysis of osteocalcin staining in **l**. *n* = 5, *P < 0.05. **n** Cofluorescence staining of CD31 (green) and endomucin (EMCN) (red) in mouse femurs. **o** Quantitative analysis of the ratio of CD31 and EMCN double-positive fluorescence intensity in **n**. *n* = 5, *P < 0.05. **p** Representative images of TRAP staining of the distal femurs. **q** Quantitative analysis of TRAP staining in **p**. The number of TRAP^+^ cells was normalized to the bone area. *n* = 5

To further investigate whether PDGF-BB secreted by TRAP^+^ mononuclear cells mediates Siglec-15 neutralizing antibody-induced bone formation, we generated PDGF-BB conditional knockout mice (*PDGFB*^*−/−*^) by crossing TRAP-Cre mice with *PDGF-BB*^*flox/flox*^ mice, in which *PDGFB* was specifically deleted in TRAP^+^ lineage cells. Adult *PDGFB*^*−/−*^ mice and their age-matched WT littermates (*PDGFB*^*f/f*^) were injected with different antibodies twice weekly for 2 months. The effect of the Siglec-15 neutralizing antibody NP-159 on bone formation was abolished in the *PDGFB*^*−/−*^ mice relative to the *PDGFB*^*f/f*^ mice. In µCT analysis, the *PDGFB*^*f/f*^ mice injected with NP-159 showed significantly higher BV/TV (Fig. 5a, b) and Tb. N values (Fig. 5a, c), as well as lower Tb. Sp values (Fig. 5a, d), relative to the *PDGFB*^*f/f*^ mice injected with NP-149. This anabolic effect was absent in the *PDGFB*^*−/−*^ mice (Fig. 5a–d). We did not observe a significant difference in cortical bone parameters among the different groups of mice except the trend of bone volume increase between the NP-159- and NP-149-treated *PDGFB*^*f/f*^ mice (Fig. 5a, e–g). Double labeling and trichrome staining showed that NP-159 treatment increased the bone formation rate in the *PDGFB*^*f/f*^ mice, as evidenced by increases in the mineral apposition rate (Fig. 5h, i), osteoid surface area (Fig. 5j, k), and number of osteoblasts (Fig. 5l, m). Again, no differences were observed in the *PDGFB*^*−/−*^ mice relative to the NP-149-treated *PDGFB*^*f/f*^ mice for these parameters (Fig. 5h–m). Coimmunofluorescence staining of CD31 and EMCN showed that the increase in type H vessels induced by NP-159 treatment was blunted in the *PDGFB*^*−/−*^ mice (Fig. 5n, o). To determine whether the effect of NP-159 treatment on osteoclastogenesis was altered by *PDGFB* knockout, we performed TRAP staining. The differences between the mice treated with NP-159 and NP-149 in the number and size of osteoclasts were similar in both the *PDGFB*^*f/f*^ and *PDGFB*^*−/−*^ mice (Fig. 5p, q). These data indicate that although the Siglec-15 neutralizing antibody blocked the maturation process of osteoclasts in both *PDGFB*^*−/−*^ mice and *PDGFB*^*f/f*^ mice, the lack of PDGF-BB secretion by TRAP^+^ mononuclear cells mediated the anabolic effect of the Siglec-15 neutralizing antibody on bone formation.

### Injection of a Siglec-15 neutralizing antibody prevented bone loss in estrogen-depleted osteoporotic mice

**Fig. 6:**
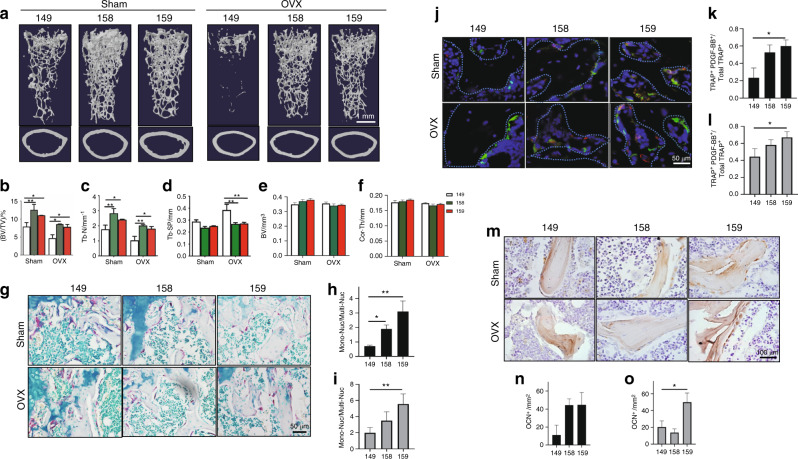
Injection of the Siglec-15 neutralizing antibody prevented bone loss in estrogen-depleted osteoporotic mice. All data in this figure are derived from 5-month-old C57BL/6 mice that underwent OVX at 3 months old and were treated with NP-149 (control antibody), NP-158 (Siglec-15 neutralizing antibody 1), or NP-159 (Siglec-15 neutralizing antibody 2) for 2 months after surgery. **a** Representative three-dimensional µCT images of the mouse femurs. Quantitative analysis of the µCT scan in **a**: **b** BV/TV percentage trabecular bone volume, **c** Tb. N trabecular number, and **d** Tb. Sp trabecular separation. **e** BV cortical bone volume, **f** Cor. Th cortical bone thickness. *n* = 5, *P < 0.05, **P < 0.01. **g** Representative images of TRAP staining of the distal femurs. Quantitative analysis of TRAP staining in the sham-operated group (**h**) and ovariectomy group (**i**). Mon-Nuc: mononuclear TRAP^+^ cells, Multi-Nuc: multinuclear TRAP^+^ cells. *n* = 5, **P < 0.01. **j** Coimmunofluorescence staining of TRAP (green) and PDGF-BB (red) in the trabecular bone of mouse distal femurs. Quantitative analysis of the ratio of TRAP/PDGF-BB double-positive fluorescent cells in TRAP^+^ cells in the sham-operated (**k**) and ovariectomy (**l**) groups. *n* = 5, *P < 0.05. **m** Representative images of immunohistological staining of osteocalcin in mouse distal femurs. Quantitative analysis of osteocalcin staining in the sham-operated (**n**) or OVX (**o**) groups. *n* = 5, *P < 0.05

To investigate the therapeutic potential of the Siglec-15 neutralizing antibody for treating bone loss, we examined the effect of Siglec-15 antibodies in an ovariectomized (OVX) mouse model in which estrogen depletion induces osteoporosis. In this experiment, we compared the effect of one Siglec-15 antibody engineered with two different Fc domains (NP-158 and NP-159) and used NP-149-treated mice as the controls. We administered the Siglec-15 antibodies NP-159, NP-158, or NP-149 twice weekly for 8 weeks to 3-month-old female C57/bl6 mice that underwent OVX or sham surgery. μCT analysis showed that OVX-induced estrogen deficiency resulted in significant bone loss in the NP-149-treated mice. This pathological change was prevented by NP-158 or NP-159 treatment. Administration of both NP-159 and NP-158 significantly increased the BV/TV and Th. N values in the sham-operated and ovariectomized mice compared with the NP-149-treated mice (Fig. 6a–c). A significant difference in Th. Sp was found between the OVX mice treated with NP-149 and those treated with NP-158 or NP-159 (Fig. 6a, d). The effect of NP-158 in increasing bone density seemed to be slightly stronger than that of NP-159, but there was no significant difference between these two groups. The effects of both NP-158 and NP-159 on cortical bone did not achieve a significant difference compared to that of the NP-149-treated groups (Fig. 6a, e, f). Similar to that in the SKO mice, the ratio of mononuclear TRAP^+^ immature osteoclasts to multinuclear mature osteoclasts in the trabecular area was significantly higher in the NP-159- and NP-158-treated sham-operated or OVX mice than in the NP-149-treated mice (Fig. 6g–i). Double immunofluorescence staining of TRAP and PDGF-BB showed that treatment with NP-158 or NP-159 increased the number of cells that were double-positive for TRAP and PDGF-BB in both the sham-operated and OVX groups; however, only the NP-159 group showed a significant difference when compared with the NP-149 group (Fig. 6j–l). As expected, the number of osteoblasts on the bone surface was higher in the mice treated with NP-158 or NP-159 than in the NP-149-treated mice (Fig. 6m–o). Our findings show that the neutralizing antibody against Siglec-15 effectively prevents estrogen depletion-induced bone loss in adult female mice.

### Injection of the Siglec-15 neutralizing antibody promoted fracture healing

**Fig. 7:**
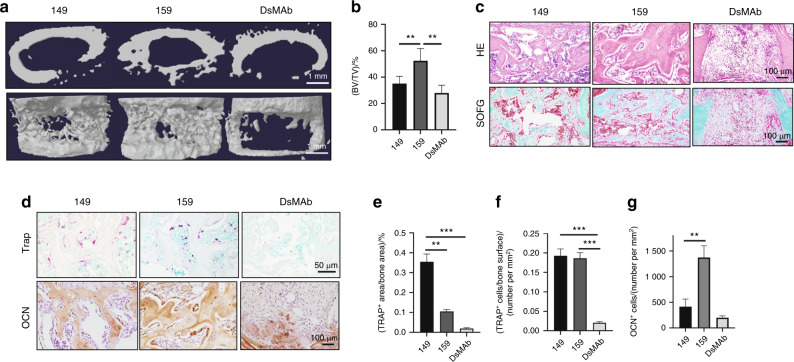
Injection of the Siglec-15 neutralizing antibody promoted fracture healing. All data in this figure are derived from 5-month-old C57BL/6 mice that underwent semiosteotomy at 3 months old and were treated with NP-149 (control antibody) or NP-159 (Siglec-15 neutralizing antibody 2) for 1 month after surgery. **a** Representative three-dimensional µCT images of the bone defect area in the mouse femurs. **b** Quantitative analysis of the µCT scan in **a**. *n* = 5, **P < 0.01. **c** Representative images of hematoxylin and eosin (HE staining (top row) and safranin O fast green (SOFG) staining (bottom row) of the bone defect in mouse femurs. **d** TRAP staining (top row) and immunohistological staining of osteocalcin (OCN) (brown, bottom row) in the bone defect area in mouse femurs. Quantitative analysis of the number (**e**) and area (**f**) of TRAP^+^ cells in the bone defect area. *n* = 5, **P < 0.01, ***P < 0.001**. g** Quantitative analysis of the number of OCN^+^ cells in the bone defect area. *n* = 5, **P < 0.01

Fracture is the major consequence of osteoporosis, and reunion of the cortical bone is the primary sign of fracture healing. Currently, no medication effectively promotes cortical bone formation. A previous study showed that the number of TRAP^+^ cells in the periosteum is significantly lower in adult mice (3 and 6 months old) than in young mice (0.5 and 1 month old).^21^ This mechanism may be the reason for the high number of nonunions in elderly patients with osteoporosis-related fractures. We tested whether the Siglec-15 neutralizing antibody can rejuvenate the bone-forming capacity of the periosteum by increasing the number of premature osteoclasts and, subsequently, PDGF-BB production. We tested our hypothesis in two types of bone healing mouse models. In the first model, a transcortical hole was created using a microdrill at the midsheaf of the femur, which mimics damage caused by the screw of an external fixator (Fig. S2). In the second model, a semiosteotomy was performed at the midshaft of the mouse femurs, which resembled the clinical fracture healing process (Fig. 7). We started the 4-week treatment immediately after surgery. In both mouse models, we compared the therapeutic effect of NP-159 with denosumab, the FDA-approved monoclonal antibody, on RANKL, and we used NP-149 as the negative control. NP-159 treatment significantly promoted bone healing in both mouse models relative to NP-149 treatment, as evidenced by the lower defect volume on μCT analysis (Fig. 7a, b) and histological analysis (Fig. 7c). Notably, denosumab did not have a beneficial effect on bone healing relative to NP-149 treatment (Fig. 7a–c). We then performed TRAP staining to detect the number, location, and morphology of immature/mature osteoclasts in the damaged area. Relative to that of the NP-149-treated mice, the size of TRAP^+^ cells in the NP-159-treated mice decreased, as indicated by the significantly decreased TRAP^+^ areas, whereas the number of TRAP^+^ cells remained comparable to that of the NP-149-treated mice. TRAP^+^ cells were rarely detected in the denosumab-treated mice (Fig. 7d–f). Immunohistological staining of OCN showed that compared with that of the NP-149-treated mice, the number of mature osteoblasts in the damaged area of the cortical bone was significantly higher in the NP-159 group but not in the denosumab group (Fig. 7d, g). These findings suggest that the Siglec-15 neutralizing antibody promotes cortical bone healing by increasing the number of mononuclear preosteoclasts in the damaged area.

## Discussion

The bone mass of the SKO mice was significantly increased, and the bone phenotype was similar to that of the *SIGLEC-15* global knockout mice.^2^ Moreover, Siglec-15 has been reported to be expressed primarily by osteoclast lineage cells and was not identified in osteoblast-lineage cells.^3^ Our osteoblast differentiation assays indicate that the bone-forming capacity of osteoblast-lineage cells is unchanged in the SKO mice. These findings suggest that the enhanced bone formation in the SKO mice was modulated indirectly by osteoclast lineage cells. Notably, deleting *SIGLEC-15* in macrophage-lineage cells (lysozyme positive) did not affect the differentiation of these cells to become TRAP^+^ osteoclastic cells, as shown by the total number of TRAP^+^ cells remaining unchanged in the SKO mice compared with the WT mice. However, the TRAP^+^ cells in the SKO mice were smaller and contained fewer nuclei, indicating that the process of multinucleation and maturation of osteoclasts is disrupted in the absence of Siglec-15.

Previous studies have investigated how Siglec-15 regulates osteoclast formation.^2^ The expression of Siglec-15 in mouse bone marrow macrophages was upregulated by RANKL. Siglec-15 contains a distinctive positively charged residue within its transmembrane domains that can interact with transmembrane immunoreceptor tyrosine-based activation motif (ITAM)-bearing coreceptors, such as DNAX-activating protein 12 kD (DAP12). By forming a complex with DNAX-activating protein 12 kD (DAP12), Siglec-15 can promote RANKL-induced osteoclast development through the ITAM domain of DAP12.^2^ Here, we show that a Siglec-15 neutralizing antibody inhibits mature osteoclast formation, yet monocytes are still able to differentiate into TRAP^+^ cells. Our data suggest that Siglec-15 is critical to the process of osteoclast fusion. Interestingly, the changes in serum markers of bone formation (OCN) and bone resorption (CTX) were not identical in the SKO mice and the Siglec-15 neutralizing antibody-treated mice relative to their respective controls. The increase in OCN in the SKO mice was not significant, while the expression was significantly increased in the NP-159-treated mice. In contrast, CTX significantly decreased in the SKO mice but not in the NP-159-treated mice. Siglec-15 was reported to induce Akt activation by interacting with DAP12.^3^ Mice lacking ITAM harboring adapters, Fc receptor common γ (FcRγ), and DAP12 exhibit severe osteopetrosis owing to impaired osteoclast differentiation.^25^ Activation of osteoclast-associated receptor/FcRγ signaling rescued impaired osteoclastogenesis in Siglec-15-deficient cells.^2^ These findings suggest that in addition to promoting osteoclast fusion, the Siglec-15 signaling pathway plays a critical role in osteoclastogenesis. It is conceivable that inhibition of bone resorption is more pronounced when Siglec-15 is completely depleted in the SKO mice than in the neutralizing antibody-treated mice. Moreover, the commitment of preosteoclasts from bone marrow monocytes may also be severely suppressed in the SKO mice, as Siglec-15 can regulate the effect of RankL on osteoclast lineage cells. As a result, the bone formation potential in the SKO mice may be relatively lower than that of the neutralizing antibody-treated mice, as the formation of mononuclear TRAP^+^ cells is likely to also be suppressed in the SKO mice.

Although the immaturity of osteoclasts may represent decreased bone resorption activity and consequently reduce the liberation of active transforming growth factor-β from the bone matrix, the PDGF-BB secreted by TRAP^+^ mononuclear cells likely overrides this negative effect. Moreover, the nuclear factor kappa-light-chain-enhancer of activated B cells (NF-κB) signaling pathway is reportedly associated with PDGF-BB production.^26,27^ A recent study showed that NF-ĸB can bind to the *Pdgfb* promoter in preosteoclasts and is necessary for the induction of PDGF-BB expression.^28^ Interestingly, Siglec-G negatively regulated the activation of NF-ĸB. It is unknown whether Siglec-15 has a similar effect to that of Siglec-G in regulating the signals of NF-ĸB. If so, antibodies against Siglec-15 may promote PDGF-BB secretion through two mechanisms. The anabolic effect of Siglec-15 neutralizing antibody in increasing bone mass would make it superior to alendronate for treating osteoporosis.

The dual effects of the Siglec-15 neutralizing antibody against osteoporosis were achieved by decreasing the number of mature osteoclasts. Fewer mature osteoclasts lead to less bone resorption, and bone formation is increased by the promotion of PDGF-BB production and subsequent type H vessel formation secondary to the increased mononuclear TRAP^+^ cells. Our theory is partially supported by the finding that v-ATPase V0 subunit-deficient mice with failure of fusion of preosteoclasts showed increased bone formation.^29^ In estrogen deficiency-induced bone loss, excessive osteoclastic bone resorption is not the only challenge; impaired bone formation, vascularization, and increased inflammation levels are also associated with OP progression.^30^ Clinical concomitant use of antiresorptive medications with teriparatide mostly failed to reverse the impairment of anabolism.^18^ In our study, the anabolic effects of the Siglec-15 neutralizing antibody were achieved mainly by enhancing vascular factor PDGF-BB and the increase of CD31^high^Emcn^high^ vessel formation, a recently characterized capillary subtype that is associated with osteogenesis.^22^ Recent studies also confirmed that the loss of type H vessels is a sensitive biomarker in human bone loss,^31^ and therapy that increases type H vessel formation was effective in reversing bone loss in a mouse ovariectomy model.^32^ Type H vessels have been shown to generate a specific microenvironment that favors bone formation by providing metabolic and molecular support as well as maintaining perivascular osteoprogenitors.^22^ RankL secreted by osteoblast-lineage cells is the key factor for osteoclastogenesis, while premature osteoclasts can promote angiogenesis by producing PDGF-BB. The presence of molecular crosstalk from osteoblast-lineage and osteoclast lineage cells to endothelial cells synergistically promotes angiogenesis and bone formation.^33^

Nonunion of bone after fracture is an orthopedic condition with a high morbidity and clinical burden, especially in the elderly population. Despite the estimated global prevalence of nine million OP-induced fractures annually, bone regeneration therapy is limited, resulting in patients living with pain, reduced quality of life, and associated psychological, social, and financial repercussions.^34^ Poor vascularization is a major cause of bone nonunion after fracture, leading to failure in blood supply and delayed healing.^35^ Currently, no efficient medication can directly promote cortical bone formation. Advances have been made in treating nonunion by improving vascularization.^36^ The molecular pathogenesis of fracture nonunion has been identified, but the temporal and spatial vascularization in fracture nonunion remains unsolved.^35^ A previous study also showed that the increase in mononuclear osteoclasts in the periosteum significantly promotes cortical bone formation by increasing type H vessel vascularization.^22^ In our study, we facilitated fracture healing, providing an alternative solution for treating fracture nonunion. Importantly, our findings also suggest that targeting Siglec-15 can promote intramembranous and endochondral ossification. Therefore, the Siglec-15 neutralizing antibody has the potential to be developed as a novel drug for treating osteoporosis and osteoporosis-induced fracture.

## Materials and methods

### Mice

All mice were maintained at the animal facility of The Johns Hopkins University School of Medicine (Baltimore, MD, USA). The procedures were performed under a protocol approved by the Institutional Animal Care and Use Committee of The Johns Hopkins University. All mice had free access to autoclaved water and a sterilized maintenance diet in a 12-h light–dark cycle at a room temperature of 21 °C ± 2 °C.

### Mouse sourcing and genotyping

LysM-Cre, PDGF-BB^f/f^, and C57BL/6 mice (referred to as “WT”) were purchased from the Jackson Laboratory (Bar Harbor, ME, USA). Siglec-15^f/f^ mice were obtained from the laboratory of L.C. (Yale University, New Haven, CT, USA). TRAP-Cre mice were obtained from the laboratory of Dr. Jolene J. Windle (Virginia Commonwealth University, Richmond, VA, USA). We generated LysMCre:Siglec-15^f/f^ (SKO) mice by crossing LysM-*Cre* mice with Siglec-15^f/f^ mice.

TRAP-Cre:PDGF-BB^f/f^ conditional knockout mice were generated by crossing TRAP-Cre mice with PDGF-BB^f/f^ mice. Genotypes were determined by polymerase chain reaction analyses of genomic DNA isolated from mouse toes using the following primers: Siglec-15 forward, 5′-CTTCAGGTGAATCACAGCTTCATGC-3′, reverse: 5′-GAAGCGTCTCTTCTAGTTTTCACAAGGG-3′; TRAP-Cre forward, 5′-ATATCTCACGTACTGACGGTGGG-3′, reverse: 5′-CTGTTTCACTATCCAGGTTACGG-3′; LoxP/PDGF-BB forward, 5′-GGGTGGGACTTTGGTGTAGAGAAG-3′ and reverse, 5′-GGAACGGATTTTGG AGGTAGTGTC-3′.

### Mouse models and treatment

#### Ovariectomy model

Eleven-week-old female C57BL/6 mice were randomized to each group (five mice per group) after acclimatization for 1 week. At 12 weeks of age, the mice were anesthetized by intraperitoneal injection of a mixture of ketamine (Vetalar, Ketaset, Ketalar; 100 mg·kg^−1^) and xylazine (Rompun, Sedazine, AnaSed; 10 mg·kg^−1^). After anesthesia, a 1.5-cm incision was made in the middle of the abdomen between the last coastal ridge and thigh. The abdominal cavity was opened under aseptic conditions, and both ovaries were exteriorized by ligating the uterine tube on both sides. The incisions of the muscle and the skin were closed using 6-0 silk sutures.

#### Bone fracture healing models

Twelve-week-old male C57BL/6 mice were anesthetized in the same manner as the ovariectomy model. The femoral condyle was exposed by patellar dislocation. In model 1, a hole (0.5-mm diameter) was made at the intercondylar notch of the femur using a microdrill. In model 2, a 0.6-mm defect in the middle of the femur was generated between the intercondylar end and the femur trochanter using a standard wire saw (0.66 mm, RISystem AG, Davos, Switzerland). After removal of bone fragments with sterile 0.01 mol·L^−1^ phosphate-buffered saline (PBS), we repositioned the patella and closed the skin incision using 6-0 silk sutures.

The treated mice were transferred to cages when they recovered from surgery (regained consciousness and normal capacity of movement) and were examined twice a day. For the ovariectomy model, the Siglec-15 neutralizing antibodies NP-159 (10 mg·kg^−1^), NP-158 (10 mg·kg^−1^), or NP-149 (10 mg·kg^−1^) were administered peritoneally three times a week for 8 weeks. For the fracture healing model, NP-149 (10 mg·kg^−1^), NP-159 (10 mg·kg^−1^), and anti-mouse RANKL (5 mg·kg^−1^) monoclonal antibodies (IK22/5, Bio X Cell, Lebanon, NH, USA) were administered peritoneally three times a week for 4 weeks.

### μCT analyses

Mice were euthanized with an overdose of isoflurane inhalation and then subjected to transcardiac perfusion with 50 mL of PBS, followed by 10% buffered formalin. The mouse femurs were isolated and fixed in 10% buffered formalin for 24–48 h, followed by high-resolution μCT scanning (SkyScan 1172, Bruker-microCT, Kontich, Belgium) under 65 kV and 153 μA with a 1.0-mm aluminum filter. The voxel size of the image was 9 μm. Reconstructive images were generated using NRecon v1.6 software (SkyScan US, San Jose, CA, USA) and analyzed using CTAn v1.9 and dataview (SkyScan US). Three-dimensional images were generated by CTVol v2.0 (SkyScan US). To determine the trabecular bone density, we analyzed the BV/TV, Tb.Th, Tb.N, and Tb.Sp of the distal femur (1.72 mm long, 0.215 mm under the growth plate). To examine bone fracture healing, we analyzed the defect size of the cortical bone.

### μCT-based angiography

We used μCT to image bone blood vessels of the whole femur by injecting Microfil silicone rubber injection compounds (MV-122, Flow Tech, Inc., Carver, MA, USA). After anesthetization, the thoracic cavity of the mice was opened. A 26-G needle was inserted into the left ventricle, and the vasculature was perfused with 50 mL of heparinized 0.01 mol·L^−1^ PBS (0.01 mol·L^−1^ PBS and 100 U·mL^−1^ heparin sodium), followed by 10 mL of 10% neutral buffered formalin as pressure fixation. Ten microliters of low-viscosity Microfil liquid (5 mL MV-compound, 4 mL MV-diluent, and 1 mL MV curing agent) was injected transcardially into the mouse vasculature immediately after mixing the reagents. Mouse bodies were stored at 4 °C for 24 h. The femur was then removed and fixed in 10% neutral buffered formalin for 24 h. After the femur was decalcified using 0.5 mol·L^−1^ ethylenediaminetetraacetic acid, we measured the vascular volume and density using μCT. The scanner was set at a voltage of 65 kV, a current of 153 μA, and a resolution of 9 μm per pixel.

### Cell culture

#### Osteoclastogenesis of primary mouse bone marrow monocytes

The hind limbs of 8-week-old mice were harvested by carefully removing the attached soft tissue. The bones (femurs and tibias) were then washed three times with PBS +10% penicillin and streptomycin. We collected bone marrow cells by cutting both ends of the tibia and femur and then flushing the marrow with a syringe using α-minimum essential medium (α-MEM). Whole bone marrow cells were collected through centrifugation for 15 min at 1 000 r·min^−1^ and then cultured in α-MEM containing 100 U·mL^−1^ penicillin (Sigma-Aldrich, St. Louis, MO, USA), 100 μg·mL^−^^1^ streptomycin sulfate (Sigma-Aldrich), and 10% fetal bovine serum (FBS) (Sigma-Aldrich) at 37 °C in a 5% CO_2_-humidified incubator. After 24 h, the nonadherent cells floating in the culture media were collected and cultured in α-MEM supplemented with M-CSF (30 ng·mL^−^^1^). After 3 days, the macrophage-lineage cells were collected by digesting the adherent cells with Gibco Versene Solution (5040066, Thermo Fisher Scientific, Waltham, MA, USA). The cells were reseeded in 6-well plates (5 × 10^5^ cells per well) and cultured in α-MEM containing 30 ng·mL^−1^ M-CSF and 100 ng·mL^−1^ RANKL (PeproTech, Cranbury, NJ, USA). Moreover, we treated the cells with NP-149 (5 μg·mL^−1^) or NP-159 (5 μg·mL^−1^) for 7 days. After treating the cells with osteoclastogenic media for 6 days, we changed the culture media to FBS-free α-MEM while the other reagents remained the same. The conditioned medium was collected 24 h later. The conditioned medium was stored at −80 °C after centrifugation at 10 000 r·min^−1^ for 15 min at 4 °C until ELISAs.

#### Staining of the osteoclasts

After the cells were treated with osteoclastogenic media for 7 days, the medium was removed, followed by fixation for 10 min using 4% paraformaldehyde. Fluorescence staining of F-actin with phalloidin was used to observe action ring formation and osteoclast polarization. A TRAP staining kit (Sigma-Aldrich) was used to detect TRAP^+^ cells according to the manufacturer’s instructions. Sample images were captured by a fluorescence microscope (BX51, DP72, Olympus Scientific Solutions Americas, Inc., Waltham, MA, USA).

#### Bone resorption test

To assess the effect of Siglec-15-induced bone resorption, we performed an osteoclast bone resorption assay using a commercially available bone resorption assay kit (Cosmo Bio Co., Ltd., Tokyo, Japan). Bone marrow monocytes of the WT and SKO mice were seeded at a density of 1 × 10^3^ cells per cm^2^ on bone slices in 24-well plates and cultured with M-CSF (30 ng·mL^−1^) for 3 days. NP-149 (5 μg·mL^−1^) or NP-159 (5 μg·mL^−1^) was added to the medium together with RANKL (100 ng·mL^−1^) in the presence of M-CSF (30 ng·mL^−1^) for 21 days. The resorption pits on the hydroxyapatite surface were imaged under a microscope and analyzed using ImageJ software (National Institutes of Health, Bethesda, MD, USA).

#### Osteoblast differentiation assay

The procedure for harvesting whole bone marrow cells was the same as described above. After culturing the cells in α-MEM containing 100 U·mL^−1^ penicillin (Sigma-Aldrich), 100 μg·mL^−1^ streptomycin sulfate (Sigma-Aldrich), and 10% FBS (Sigma-Aldrich) at 37 °C in a 5% CO_2_-humidified incubator for 24 h, we cultured the adherent cells in Dulbecco’s modified Eagle’s medium (Sigma-Aldrich) for an additional 7 days with 10% FBS. Then, the cells that formed clones were digested and reseeded in 6-well plates at a density of 5 × 10^4^ cells per cm^2^. We performed crystal violet staining and Alizarin red staining as previously described. NP-149 (5 μg·mL^−1^) or NP-159 (5 μg·mL^−1^) was added to the medium as indicated. After 10 days of culture at 37 °C in a 5% CO_2_-humidified incubator, the formation of cell clones was evaluated. The cultures were terminated and washed with PBS, fixed with 4% paraformaldehyde for 10 min, and stained with 0.5% crystal violet in methanol for 10 min at room temperature. We counted the colonies that contained 50 cells or more. For Alizarin red staining, the cells were cultured in complete media prepared with the MesenCult mouse Osteogenic Stimulatory Kit (StemCell Technologies; Vancouver, BC, Canada). Then, the cultures were terminated at 21 days of culture, washed with PBS twice, and fixed with 4% paraformaldehyde. We evaluated cell-matrix mineralization by Alizarin red S staining (2% Alizarin red S, Sigma-Aldrich) dissolved in distilled water with the pH adjusted to 4.2. Colonies that stained positive with Alizarin red S were counted. The results of the CFU‐F and CFU‐OB assays are reported as the mean ± standard deviation of triplicate cultures.

### ELISA and western blots

We obtained whole blood samples from the right ventricle of mice by cardiac puncture immediately after euthanasia. Serum was collected by centrifuging blood samples at 200 × *g* for 15 min. The bone marrow supernatant was collected by cutting both ends of the femur. The samples underwent centrifugation at 3 000 r·min^−1^ and 4 °C for 15 min. The collection of conditioned media has been described above. The cell culture supernatants were collected by centrifugation at 200 × *g* for 15 min. All samples were stored at −80 °C before analyses. The concentration of PDGF-BB in the bone marrow, serum, and cell culture supernatants was determined using the PDGF-BB Assay Kit (MBB00, R&D Systems, Minneapolis, MN, USA) according to the manufacturer’s instructions (three per group).

Western blot analysis was conducted using cell lysates. The cell lysates were centrifuged, and the supernatants were separated by sodium dodecyl sulfate–polyacrylamide gel electrophoresis and then blotted on polyvinylidene fluoride membranes (Bio-Rad Laboratories, Hercules, CA, USA). Specific antibodies were applied for incubation, and the proteins were detected by using an enhanced chemiluminescence kit (Thermo Fisher Scientific, 46641). The antibodies used for Western blotting were PDGF-BB (1:1 000 ab23914, Abcam, Cambridge, United Kingdom) and GAPDH (1:1 000 5174, Cell Signaling Technology, Danvers, MA, USA).

### Immunohistochemistry, immunofluorescence, and histomorphometry

All tissues were fixed in 10% buffered formalin solution for 24–48 h. The femur specimens were soaked in 0.5 mol·L^−1^ ethylenediaminetetraacetic acid solution in a constant-temperature shaker at 4 °C for 3 weeks. The decalcification solution was changed once every 2 days. Samples were processed for paraffin embedment (4-μm thick sections) or cryosection (40-μm thick sections). For cryosections, samples were cryoprotected in 30% sucrose overnight at 4 °C before embedment in Tissue-Tek O.C.T. Compound (4583, Sakura Finetek, Torrance, CA, USA). The first section was determined when the anterior of the femoral condyle appeared. The last section was determined when the cortical bone of the femoral condylar disappeared. The slides were numbered to indicate the depth of the sections, and slides with similar numbers from different groups were chosen to ensure that similar anatomical levels were compared. The paraffin-embedded tissue sections were stained with hematoxylin (7231, Thermo Fisher Scientific), eosin (7111, Thermo Fisher Scientific), safranin O, and fast green stain for histological observation. Osteoclasts were detected by TRAP staining according to the manufacturer’s instructions (387A, Sigma-Aldrich). Immunostaining was performed using the standard protocol. The sections were washed three times in Tris-buffered saline Tween-20 for 5 min and permeabilized with 0.3% Triton-X (Sigma-Aldrich) for 15 min. Then, the slides were blocked with 3% bovine serum albumin (W/V) in PBS for 60 min and incubated in primary antibodies against EMCN (1:50, sc-65495, Santa Cruz Biotechnology, Inc., Dallas, TX, USA), CD31 (1:200, MAB33871, R&D Systems), TRAP (1:200, ab185716, Abcam), PDGF-BB (1:200, ab34914, Abcam), or OCN (1:20, M188, TaKaRa Bio, Shiga, Japan) overnight at 4 °C in a humidified chamber. On the second day, the sections were incubated in the appropriate secondary antibody conjugated with fluorophore or horseradish peroxidase (Jackson ImmunoResearch Laboratories, West Grove, PA, USA) to detect fluorescent signals or develop with DAB. DAPI (Vectashield H-1500, Vector Laboratories, Inc., Burlingame, CA, USA) or hematoxylin counterstaining was performed to label the nuclei in immunofluorescence staining or immunohistological staining, respectively. We used an Olympus BX52 microscope (Olympus Scientific Solutions Americas, Inc.) or a Zeiss LSM 780 confocal microscope (Carl Zeiss Microscopy, LLC, White Plains, NY, USA) for sample image capture. ImageJ software (National Institutes of Health) was used for quantitative analysis.

### Double-labeling analysis

A standard double-labeling procedure was performed to examine dynamic bone formation. We subcutaneously injected 0.1% calcein (C0875, Sigma-Aldrich) in PBS at a concentration of 10 mg·kg^−1^ at 8 days and 3% xylenol orange (90 mg·kg^−1^, 52097, Sigma-Aldrich) at 2 days before the mice were euthanized. We observed calcium double labeling in plastic embedded bone slices under a fluorescence microscope. The distance between the green fluorescence-labeled calcium layer and the red fluorescence-labeled calcium layer in three randomly selected visual fields of the distal metaphysis of the femur was measured to evaluate trabecular bone formation.

### Statistical analysis

All data analyses were performed using SPSS, version 15.0, software (IBM Corp., Armonk, NY, USA). For comparisons between the two groups, we used two-tailed Student’s *t* tests. For comparisons among multiple groups, we used one-way analysis of variance. All experiments were repeated at least three times. The difference was considered significant at *P* < 0.05.

## Supplementary information

S1

S2

## Data Availability

The control antibody NP-149, and the Siglec-15 neutralizing antibodies NP-158 and NP-159 were obtained from NextCure, Inc.
