# Supplementary figures and images for: An antibody against Siglec-15 promotes bone formation and fracture healing by increasing TRAP+ mononuclear cells and PDGF-BB secretion
Source: Bone Res. 2021 Nov 1;9:47. doi: 10.1038/s41413-021-00161-1 (PMC8558327; doi:10.1038/s41413-021-00161-1)

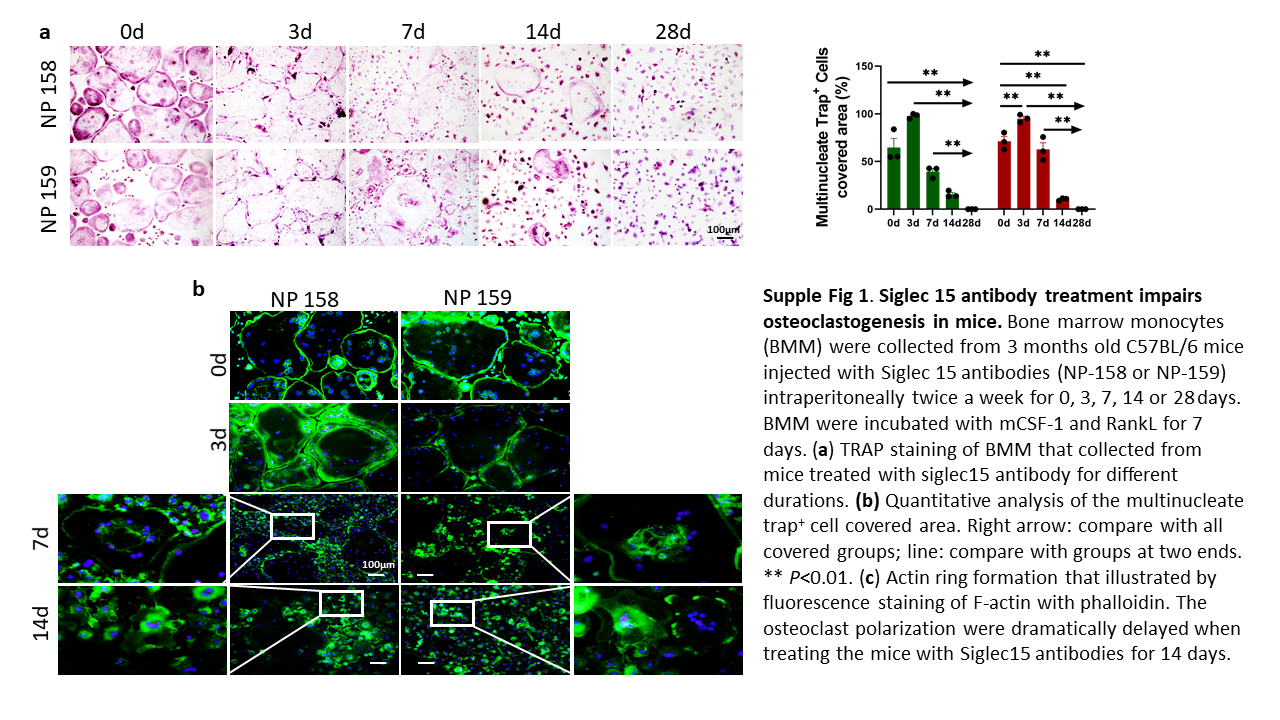

Supplement: Supplementary file 1 — S1 [file 41413_2021_161_MOESM1_ESM.tif]

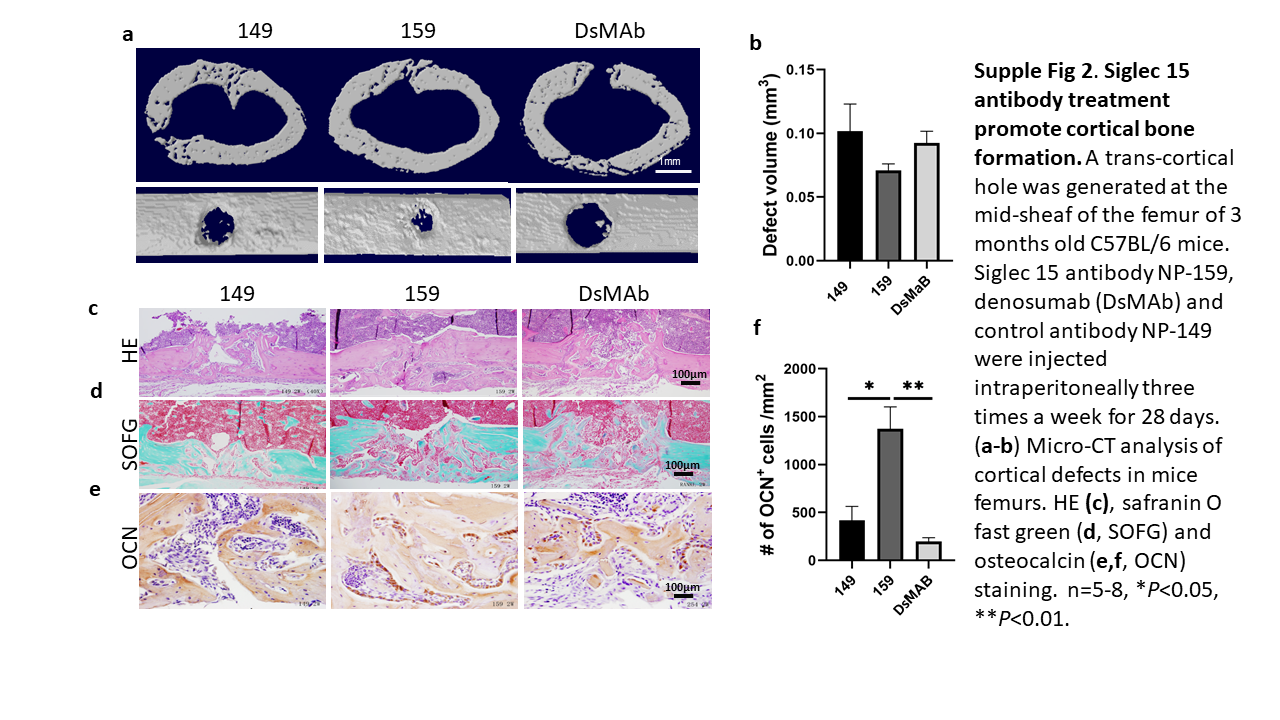

Supplement: Supplementary file 2 — S2 [file 41413_2021_161_MOESM2_ESM.tif]
